# Role of microRNA-335 carried by bone marrow mesenchymal stem cells-derived extracellular vesicles in bone fracture recovery

**DOI:** 10.1038/s41419-021-03430-3

**Published:** 2021-02-04

**Authors:** Haifeng Hu, Dong Wang, Lihong Li, Haiyang Yin, Guoyu He, Yonghong Zhang

**Affiliations:** 1 Department of Orthopaedics, Second Hospital of Shanxi Medical University, Taiyuan, Shanxi China; 2 Department of Clinical Laboratory, China-Japan Union Hospital of Jilin University, Changchun, Jilin China

**Keywords:** Stem cells, Adult stem cells

## Abstract

Mesenchymal stem cells (MSCs) have the potential to reduce healing time and treat nonunion in fracture patients. In this study, bone marrow MSCs-derived extracellular vesicles (B-EVs) were firstly extracted and identified. CD9^−/−^ and normal mice were enrolled for the establishment of fracture models and then injected with B-EVs. Osteoblast differentiation and fracture recovery were estimated. The levels of osteoblast-related genes were detected, and differentially expressed microRNAs (miRs) in B-EVs-treated normal fracture mice were screened and verified. The downstream mechanisms of miR were predicted and assessed. The loss-of functions of miR-335 in B-EV and gain-of-functions of VapB were performed in animal and cell experiments to evaluate their roles in bone fracture. Collectively, B-EVs promoted bone fracture recovery and osteoblast differentiation by releasing miR-335. miR-335 downregulation in B-EVs impaired B-EV functions in fracture recovery and osteoblast differentiation. miR-335 could target VapB, and VapB overexpression reversed the effects of B-EVs on osteoblast differentiation. B-EV treatment activated the Wnt/β-catenin pathway in fracture mice and osteoblasts-like cells. Taken together, the study suggested that B-EVs carry miR-335 to promote bone fracture recovery via VapB and the Wnt/β-catenin pathway. This study may offer insights into bone fracture treatment.

## Introduction

Fractures are the most common traumatic injuries to humans^1^, and almost occur on subcondylar, parasymphyseal, joints, and mandibular, pelvis and third metacarpal bone^2,3^. In children, abuse is the main contributor to femur fracture, and motor vehicle accidents are the most common cause of femur fracture for adults, whereas in the elderly, femur fractures almost result from falling^4^. Patients with Parkinson’s disease, stroke, or heart failure have a high risk of hip and femur fracture, and they are more likely to die or experience prolonged disability when a fracture occurs^5,6^. Although autogenous and vascularized bone transplantation has been used to treat delayed union or nonunion fractures, the current treatment is often costly and ineffective^7^. Besides, fracture recovery involves the synergistic action of stem cells mainly from periosteum, and these stem cells differentiate into chondrocytes and osteoblasts, forming cartilage callus, and then bone callus^8^. In addition, studies have confirmed that adequate amount of mesenchymal stem cells (MSCs), especially bone marrow-derived MSCs (BMSCs) and the microenvironment around the fracture are effective for osteogenic differentiation and fracture repair^9,10^. Therefore, it is of prime importance to substantially reduce the incidence and consequences of fractures by reaching more BMSCs-mediated therapies.

Evidence has indicated MSCs are attractive to regenerative medicines due to their abilities in self-renewal, regeneration of damaged tissues and multilineage differentiation, and immunosuppressive capacity in regulating autoimmune diseases^11^. It has been reported so far that MSCs could secrete several kinds of extracellular vesicles (EVs)^12^. EVs are endocytic membrane-derived vesicles released by cells, which could mediate intercellular communication by carrying proteins, microRNAs (miRs), and RNAs between cells or remote organs^13^. Importantly, in the bone microenvironment, EVs also play an important role in the communication between bone cells, namely osteoblasts, osteoclasts, and BMSCs^14^. More and more attention has been paid to the application of stem cell-derived EV therapy in osteogenesis and fracture healing^15^. MSCs-derived EVs saved the delay of fracture healing in CD9^−/−^ mice and wild-type (WT) mice^7^. In addition, it has been suggested that a large number of miRs participate in EV-mediated intercellular communication and play significant roles in morphogenesis, tissue repair, and organogenesis^16^. Here, in this study, we firstly explored the dysregulated miRs in BMSCs-derived EVs (B-EVs), and then studied the effects of B-EVs and the molecular mechanisms in fracture recovery.

## Materials and methods

### Ethics statement

This study was approved by the Clinical Ethical Committee of Second Hospital of Shanxi Medical University. All animal experiments were conducted with the approval of the Institutional Animal Care and Use Committee. Great efforts were made to minimize the animals and their pains.

### Isolation and identification of EVs

Murine BMSCs were obtained from Shanghai Institute of Cellular Biology of Chinese Academy of Sciences (Shanghai, China) and cultured in MSC growth medium (Lonza Group Ltd., Basel, Switzerland) at 37 °C with 5% CO_2_. BMSCs of the 4th–6th generation were used for subsequent experiments. BMSCs were identified according to the method reported in the previous literature^17^. The extraction of BMMSC derived extracellular vesicles (B-EVs) was carried out according to the previous reports^7,18^. BMSCs were seeded in six-well plates with MSC growth medium at a density of 1.03 × 10^5^/well. After 1 day of culture, the cells were washed with phosphate-buffered saline (PBS) three times, and the medium was replaced by EVs-free medium for 48 h. Then, 2 mL conditioned medium (CM) was collected and centrifuged at 300 × *g* and 4 °C for 10 min to remove cells, then centrifuged at 2000 × *g* for 10 min to remove cell debris, and then centrifuged at 10,000 × *g* for 35 min. Next, the supernatant was filtered by 0.22 μm filtration membrane (Steritop™ Millipore, Burlington, MA, USA) and centrifuged at 180,000 × *g* for 70 min. The precipitate was resuspended in PBS and centrifuged at 180,000 × *g* for 70 min. The precipitate was finally resuspended with 100 μL PBS. Bicinchoninic acid protein detection kit (Beyotime Biotechnology Co., Ltd, Shanghai, China) was used for quantitative analysis of EV protein in subsequent experiments. At the same time, an inhibitor of EV secretion GW4869 (20 μg/mL; Sigma-Aldrich, Merck KGaA, Darmstadt, Germany) was added to the medium without EV serum. After 48 h of culture, the supernatant was taken as the methods of EV isolation as the negative control (NC). The B-EVs were then identified using nanoparticle tracking analysis (NTA, Malvern, Chester County, PA, USA), transmission electron microscope (TEM) and western blot analysis. The diameter of murine B-EVs was analyzed by using NTA. The selected concentration of the samples was 1–9 × 10^8^ cells/mL, and the appropriate gray background was selected by the software and the movement of the particles was recorded. The distribution of concentration and variation of the substitute sample was drawn.

### Bone fracture model in mice

Male C57BL/6 WT mice (12 weeks old) were obtained from Hunan SJA Laboratory Animal Co., Ltd., (Changsha, Hunan, China). CD9^−/−^ mice^19^ on C57BL/6 genetic background have been previously described and propagated in our experimental facility. The mice were numbered according to the weight from light to heavy, and any number was selected from the random number table, and the mice were randomly divided into groups according to the corresponding arrangement of animal number and random number table.

The establishment of mouse fracture model was carried out according to a previous literature^7,20^. In short, mice were anesthetized with pentobarbital sodium (60 mg/kg), and a 5 mm longitudinal incision was made between the right knee joint and the hip joint under sterile conditions. The femoral shaft of one side of the exposed mouse femur was blunt separated, and the femur in the middle of the femoral shaft was cut off. Then, a 25 G syringe needle was used as an intramedullary fixation device to retrograde insert into the femoral marrow cavity and reduce the fracture end until the proximal femur was inserted. The end of the needle was cut and the incision was sutured. After that, WT mice and CD9^−/−^ mice were respectively allocated into WT + NC group (WT mice injected with EV-NC after GW4869 intervention), WT + EVs group (WT mice injected with B-EVs), WT + EVs-Mock group (WT mice injected with B-EVs prior transfected with miR-335 Inhibitor NC), WT + EVs-Inhibitor group (WT mice injected with B-EVs prior transfected with miR-335 Inhibitor), CD9^−/−^ + NC group (CD9^−/−^ mice injected with EV-NC after GW4869 intervention), CD9^−/−^ + B-EVs group (CD9^−/−^ mice injected with B-EVs), CD9^−/−^ + EVs-Mock group (CD9^−/−^ mice were injected with B-EVs prior transfected with miR-335 Inhibitor NC), CD9^−/−^ + EVs-Inhibitor group (CD9^−/−^ mice injected with B-EVs prior transfected with miR-335 inhibitor). All groups of mice were injected with 100 μL EVs purified from BMSC after different treatments or 100 μL EV-NC after GW4869 treatment at the fracture site on the 1st and 8th days after fracture. miR-335 inhibitor and its NC were purchased from GenePharma (Shanghai, China). According to the instructions of Lipofectamine 2000 (Invitrogen, Carlsbad, CA, USA), miR-335 inhibitor and its NC were transfected into BMSC for 48 h with a final concentration of 50 nM.

At 0, 1, 2, and 4 weeks after operation, the mice were anesthetized for X-ray imaging. The bone union of the fracture site was evaluated according to the X-ray scoring system described by Lane and Sandhu^21^. All imaging results were observed and analyzed by two doctors at a double-blind manner, and the values obtained were included in statistical analysis, including 0: nonunion of fracture; 1: formation of callus; 2: partial healing; 3: disappearance of fracture line; 4: complete healing. Two weeks after fracture, the fractured femur was collected for the following experiments. In the process of the experiment, blind selection method was used to evaluate the experimental results, that is, in the process of evaluation and analysis, the experimenters did not know the treatment plan of each group of mice.

### Histological evaluation

The femurs of WT and CD9^−/−^ mice were harvested 2 weeks after fracture, and fixed in 4% paraformaldehyde for 24 h. Next, the femurs were decalcified in 10% ethylene diamine tetraacetic acid (Wako, Osaka, Japan) at room temperature for 14 days and embedded in paraffin. The femurs were cut into 4.5-mm sections along the longitudinal axis with a slicer. Toluidine blue (Sigma-Aldrich), hematoxylin and eosin (HE) staining (Sigma-Aldrich), and immunohistochemistry of bone morphogenetic protein 2 (BMP2) were performed for histological difference analysis.

### Reverse transcription-quantitative polymerase chain reaction (RT-qPCR)

Total RNA from tissues and cells was acquired using the RNAiso Plus and TRIzol LS Reagent (both from Takara, Otsu, Shiga, Japan). Then formaldehyde denaturation electrophoresis was used to verify the reliability of the obtained RNA. RT-qPCR was conducted as per the manufacturer’s protocol using PrimeScript™ RT reagent kit (Takara). Finally, the mRNA expression was quantified by standard real-time qPCR protocol via SYBR Premix Ex Taq (Takara) with glyceraldehyde-3-phosphate dehydrogenase as an internal reference. The primer sequences were shown in Table 1.

**Table 1:** Primer sequences of RT-qPCR.

Primers	Forward	Reverse
miR-335	GTCGTATCCAGTGCAGGGTCCG	GTGCAGGGTCCGACCT
miR-136	ACUCCAUUUGUUUUGAUGAUG	CGTTAGACAGCCTCTTGGGG
miR-125a	GGCACTTTTCAGAACATC	TGTCGTGTATCACAGCAT
miR-217	CGGGAACAGGGCAACATCTA	TGTGTCCCTTCTTTCTGC
miR-487a	CGCTGGCAATCATACAGGGACA	GTGCAGGGTCCGAGGT
miR-339	GGGTCCCTGTCCTCCA	TGCGTGTCGTGGAGTC
miR-298	TCAGGTCTTCAGCAGAAGC	TAGTTCCTCACAGTCAAGGA
miR-133a	UUUGGUCCCCUUCAACCAGCU	GCUGGUUGAAGGGGACCAAAUU
OCN	CTGACCTCACAGATCCCAAGC	TGGTCTGATAGCTCGTCACAAG
GDF-10	CCTGAAGGTGGATTTTGCAG	CTGACGATGCTCTGGATGG
FGF-2	CCGCCCTGCCGGAGGATGGAG	GCCTTCTGCCCAGGTCCTGT
U6	CTCGCTTCGGCAGCACA	AACGCTTCACGAATTTGCGT
GADPH	CGGACCAATACGACCAA	AGCCACATCGCTCAGACACC

Note: *RT-qPCR* reverse transcription-quantitative polymerase chain reaction, *miR* microRNA, *OCN* osteocalcin, *GDF-10* growth differentiation factor-10, *FGF-2* fibroblast growth factor-2, *GADPH* glyceraldehyde-3-phosphate dehydrogenase.

### Western blot analysis

The total proteins were extracted by radio-immunoprecipitation assay lysis buffer containing phenylmethylsulfonyl fluoride (Beyotime Biotechnology Co., Ltd, Shanghai, China) and protein level in the supernatant was then determined using the BCA method. Equal protein samples (50 mg) were loaded onto 10% sodium dodecyl sulfate–polyacrylamide gel electrophoresis and then transferred onto polyvinylidene fluoride (PVDF) membranes (Millipore, Billerica, MA, USA). After that, PVDF membranes were incubated at room temperature with tris-buffered saline tween (Boster Biological Technology Co., Ltd, Wuhan, Hubei, China) containing 5% skim milk to block nonspecific binding. Subsequently, the membranes were cultured with primary antibodies at 4 °C overnight, and then with secondary antibodies for 1 h at room temperature. At last, the proteins were colored in enhanced chemiluminescence reagent, and visualized with BioSpectrum gel imaging system (Bio-Rad, Hercules, CA, USA). All antibodies were shown in Table 2.

**Table 2:** Antibodies for western blot assay.

Antibody	No., company	Dilution ratio
CD63	ab217345, Abcam	1:1000
CD9	ab92726, Abcam	1:2000
Calnexin	ab22595, Abcam	1:2000
α-SMA	ab5694, Abcam	1:1000
OCN	AB10911, Sigma-Aldrich	1:1000
GDF-10	ab235005, Abcam	1:1000
FGF-2	05-118, Sigma-Aldrich	1:1000
VapB	ab241298, Abcam	1:2000
GAPDH	ab8245, Abcam	1:1000

### Microarray analysis

All RNA samples were submitted to Exiqon for miR profiling using the miRCURY LNA™ Universal RT microRNA PCR Mouse&Rat panel I containing 372 miRs (Exiqon, Vedbaek, Denmark). The miR raw data were background filtered and normalized.

### Culture and treatment of osteoblast-like cells

MC3T3 and MG63 cells purchased from Cell bank of Type Culture Collection Committee of Chinese Academy of Sciences (Shanghai, China) were cultivated in α-modified eagle’s medium supplemented with 10% fetal bovine serum, 100 μg/mL streptomycin, and 100 U/mL penicillin at 37 °C with 5% CO_2._ MC3T3 and MG63 cells were incubated with 100 μL EVs extracted from BMSC after different treatments for 24 h, and the control (NC) of B-EVs was the EVs from the CM after GW4869 intervention, and cells were assigned into MC3T3 + NC group (NC-treated MC3T3 cells), MC3T3 + B-EVs group (MC3T3 cells treated with B-EVs), MC3T3 + B-EVs-Mock group (BMMSC was transfected with miR-335 Inhibitor mock for 48 h, and the EVs were extracted and treated with MC3T3 cells), MC3T3 + B-EVs-Inhibitor group (BMMSC was transfected with miR-335 Inhibitor for 48 h, and the EVs were extracted and treated with MC3T3 cells), MG63 + NC group (NC-treated MG63 cells), MG63 + B-EVs group (MG63 cells treated with B-EVs), MG63 + B-EVs-Mock group (BMMSC was transfected with miR-335 Inhibitor mock for 48 h, and the EVs were extracted and treated with MG63 cells), MG63 + B-EVs-Inhibitor group (BMMSC was transfected with miR-335 Inhibitor for 48 h, and the EVs were extracted and treated with MG63 cells).

At the same time, we upregulated the expression of VapB in MC3T3 and MG63 cells treated with B-EVs. We added VapB expression plasmid with final concentration of 50 nM or empty vector into MC3T3 and MG63 cells according to the instructions of Lipofectamine 2000. The cells were assigned to MC3T3 + B-EVs + vector group (empty vector was transfected into MC3T3 cells treated with B-EVs), MC3T3 + B-EVs-inhibitor + VapB group (B-EVs treated MC3T3 cells were transfected with VapB expression plasmid), MG63 + B-EVs + vector (empty vector was transfected into MG63 cells treated with B-EVs), and MG63 + B-EVs + VapB group (B-EVs-treated MG63 cells were transfected with VapB expression plasmid).

### 3-(4, 5-dimethylthiazol-2-yl)-2, 5-diphenyltetrazolium bromide (MTT) assay

Cell activity was detected using a MTT kit in strict accordance with the instructions^22^.

### TUNEL staining

Apoptosis was detected by a TUNEL kit in strict accordance with the instructions^23^.

### Alizarin red staining

The differentiation of osteoblasts-like cells was detected by alizarin red staining according to a previous literature report^24^.

### Alkaline-phosphatase (ALP) staining

ALP activity was detected using an ALP Kit (P0321, Beyotime Biotechnology Co., Ltd, Shanghai, China) according to the previous literature report^25^.

### Immunofluorescence staining

Cells in each group on glass cover slide were rinsed with PBS three times, fixed with 4% paraformaldehyde for 30 min, and treated with 0.5% Triton-100× for 20 min. After that, sections were incubated with antibodies against F-actin (1:200, AB6016, Sigma-Aldrich), and Col I (1:200, ab96723, Abcam) at 4 °C overnight. Next, cell slides were washed with PBS, and sections were incubated with Alexa Fluora 594 or FITC-labeled goat anti-rabbit secondary antibody (1:5000, ab150088, ab6717) at 37 °C for 1 h. Later, nuclei were counterstained with 4’,6-diamidino-2-phenylindole, and cells were observed under the fluorescence microscope (DM 3000, Leica, Solms, Germany). All antibodies were provided by Abcam Inc.

### Dual-luciferase reporter gene assay

Based on bioinformatics, we predicted a binding site between miR-335 and VapB. RiboBio Biology (Guangzhou, China) was entrusted to amplify the complementary binding sequence and mutation sequence of miR-335 with VapB and cloned it into vectors to construct WT and mutant plasmids VapB-WT and VAPB mutated type. According to the instructions of Lipofectamine 2000, the plasmids were co-transfected with mimic NC or mimic miR-335 (GenePharma) respectively into HEK293T cells (Shanghai Institute of Cell Biochemistry, Chinese Academy of Sciences). The binding relationship between miR-335 and VapB was verified using Dual-Glo^®^ dual-luciferase reporter gene detection system (E2920, Promega Corporation, Madison, WI, USA)^26^.

### RNA pull-down assay

RNA-protein interactions were tested as the Pierce™ Magnetic RNA-Protein Pull-Down Kit as instructed (20164, ThermoFisher Scientific, Waltham, MA, USA).

### Statistical analysis

Statistical analysis was conducted by SPSS21.0 (IBM Corp. Armonk, NY, USA). Kolmogorov–Smirnov test checked whether the data were normally distributed. The measurement data were exhibited in mean ± standard deviation. The *t* test was applied for comparisons between two groups, while one-way or two-way analysis of variance (ANOVA) for multi-groups, and Tukey’s multiple comparisons test for pair-wise comparisons after ANOVA analyses. The *p* value was obtained by two-tailed tests and *p* < 0.05 meant statistical differences.

## Results

### Identification of BMSCs and B-EVs

Murine BMSCs were able to be induced toward osteogenic and lipogenic differentiation, and flow cytometry verified that the cells used in our experiment were in line with the definition of BMSCs (Supplementary Fig. 1A). Then, we extracted the EVs according to the steps in Supplementary Fig. 1B, and detected the size, shape, and specific protein of the EVs using the NTA, TEM, and western blot analysis. The results showed that EV size was about 120 nm, and EVs were spherical or ellipsoidal under the TEM, and CD63 and CD9 were both positive; however, no secretion of EVs was found in the extraction after GW4869 intervention; at the same time, adding PBS to the medium of BMSC had no effect on EVs (Supplementary Fig. 1C–E). These results indicate that we have successfully isolated B-EVs.

### B-EV treatment promotes the recovery of fracture in mice

**Fig. 1:**
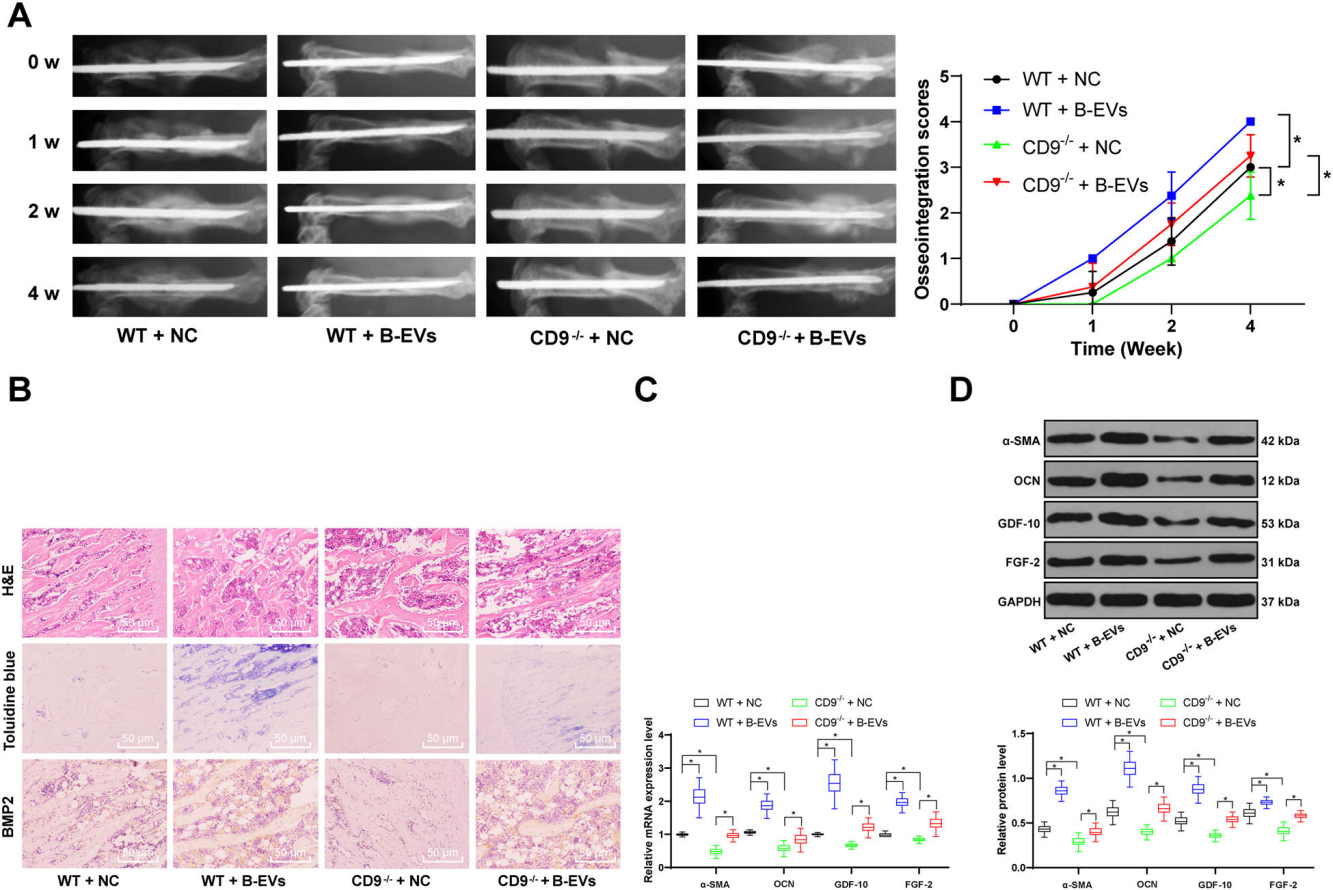
B-EV treatment promotes bone fracture recovery. **A** Representative radiologic images of WT and CD9^−/−^ fracture mice after EV treatment at 0, 1, 2, and 4 weeks after EV treatment. The femurs of WT mice and CD9^−/−^ mice were harvested 2 weeks after fracture. **B** HE staining, toluidine blue staining, immunochemistry of BMP2 were performed to access bone fracture healing. **C** RT-qPCR, and **D** western blot analysis were performed to determine mRNA and protein levels of α-SMA, OCN, GDF-10, and FGF-2. Data are expressed as mean ± standard deviation. Two-way ANOVA and Tukey’s multiple comparisons test were used to determine statistical significance. *p < 0.05. *n* = 8 in each group.

CD9 is a transmembrane protein that is involved in angiogenesis, signal transduction, cell fusion, fertilization, osteoclast formation, and myogenesis through cell adhesion and migration^27–30^. CD9 deficiency resulted in a significantly lower fracture healing rate than wild-type mice (E). At the same time, studies have also shown that CD9^−/−^ mice have reduced EV secretion after fracture^31,32^. Therefore, we speculate that the healing of fracture is related to the secretion of external vesicles. In order to test the effect of B-EVs on fracture recovery, we established fracture models of WT mice and CD9^−/−^ mice. We used X-ray to detect the recovery of fracture in mice at 0, 1, 2, and 4 weeks, respectively. It was found that no matter in WT mice or CD9^−/−^ mice, B-EV treatment was beneficial for fracture recovery, and the fracture recovery of WT mice was better than that of CD9^−/−^ mice (Fig. 1A). To evaluate the changes in the process of fracture recovery in each group, we selected the fracture tissues of mice with obvious bone healing 2 weeks after fracture. The tissues of mice in each group were extracted and subjected to HE staining, toluidine blue staining, and immunohistochemistry of BMP2. The HE staining showed that B-EV treatment promoted the formation of bone tissues, toluidine blue staining exhibited that and B-EV treatment increased cartilage tissues, and immunohistochemistry of BMP2 indicated that B-EV treatment promoted osteoblast differentiation and fracture recovery; the therapeutic effect of EVs on CD9^−/−^ mice was significantly lower than that in WT mice (Fig. 1B). After that, RT-qPCR and western blot analysis were applied to verify the mRNA and protein levels of osteogenesis-related genes. It was consistently found that B-EV increased levels of α-SMA, OCN, GDF-10, and FGF-2 (Fig. 1C–D).

### B-EVs carry miR-335 to promotes fracture recovery in mice

**Fig. 2:**
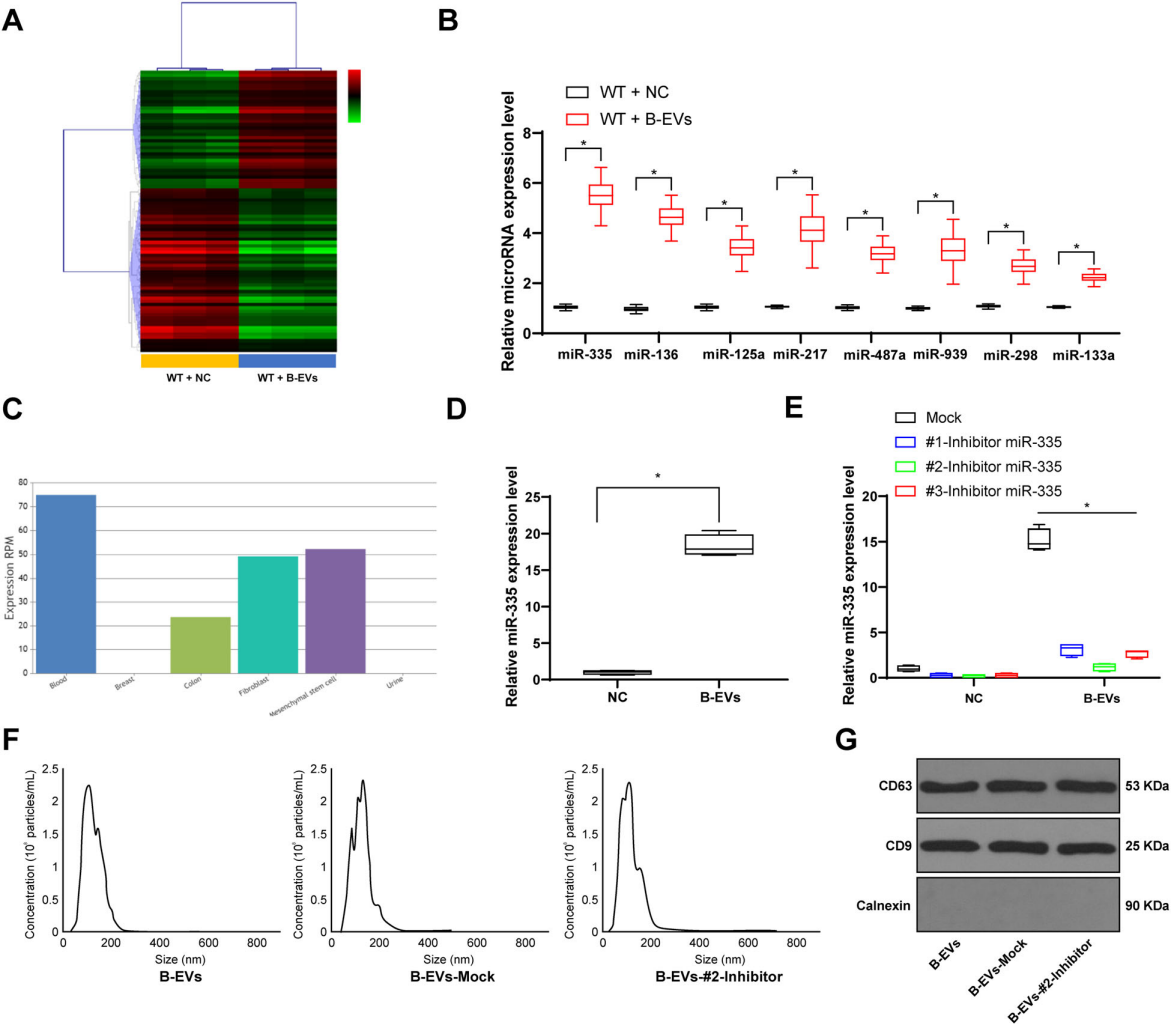
B-EV-carried miR-335 promotes bone fracture recovery. **A** Microarray assay was performed to find out the differentially expressed miRs in femurs of WT mice after B-EV treatment by miRCURY LNA™ Universal RT microRNA PCR mouse and rat. **B** RT-qPCR was performed to determine expression of miR-335, miR-136, miR-125a, miR-217, miR-487a, miR-339, miR-298, and miR-133a in femurs of WT mice to validate microarray data. **C** EvimicroRNA predicted miR-335 distribution. **D** RT-qPCR was performed to determine miR-335 expression in the conditioned medium after GW4869 intervention and EVs. **E** RT-qPCR was performed to determine miR-335 expression after miR-335 Mock or inhibitor was transfected. **F** The concentration and particle size of B-EVs transfected with 2#-Inhibitor were measured by NTA. **G** Western blot was used to detect the expression of CD63, CD9, and Calnexin on the surface of B-EVs transfected with 2#-Inhibitor. Data are expressed as mean ± standard deviation. Two-way ANOVA and Tukey’s multiple comparisons test were used to determine statistical significance. *p < 0.05. Four independent experiments were performed.

To further determine the specific mechanism of EV treatment in fracture recovery, we randomly selected three WT + PBS-treated mice and three WT + B-EV-treated mice, and extracted their cartilage tissues for microarray analysis. Compared with the WT mice, 73 miRs were downregulated and 104 miRs were upregulated after EV treatment (Fig. 2A). We screened eight differentially expressed ones, miR-335, miR-136, miR-125a, miR-217, miR-487a, miR-339, miR-298, and miR-133a for subsequent experiments. We extracted the fracture tissues of WT mice for RT-qPCR detection to verify the expression of most differentially expressed miRs. The results were consistent with those in microarray analysis, and the expression of miR-335 was the highest among eight miRs (Fig. 2B). Next, the distribution of miR-335 was analyzed on the EvimiRNA online prediction website (http://bioinfo.life.hust.edu.cn/EVmiRNA/#!/). It was found that miR-335 was abundant in MSCs (Fig. 2C). We speculated that miR-335 may play a role in the repair of fracture. RT-qPCR was then used to detect the expression of miR-335 in CM after GW4869 intervention and B-EVs, and noticed that miR-335 was abundant in B-EVs (Fig. 2D). In order to verify the effect of miR-335 on fracture recovery, we transfected miR-335 inhibitor into BMSCs and then extracted EVs. It was found that three inhibitors of miR-335 had no significant effect on the secretion of B-EVs. RT-qPCR showed that the inhibition of miR-335 by 2#-Inhibitor was the best (Fig. 2E). At the same time, in order to explore the effect of 2#-Inhibitor on the secretion of EVs, we measured the particle size of EVs transfected with 2#-Inhibitor by NTA, and detected the expression of CD63 and CD9 by western blot analysis. We found that 2#-Inhibitor had no significant effect on the secretion of B-EVs (Fig. 2F–G).

### Silencing miR-335 partially counteracts the promoting effect of B-EVs on fracture recovery

**Fig. 3:**
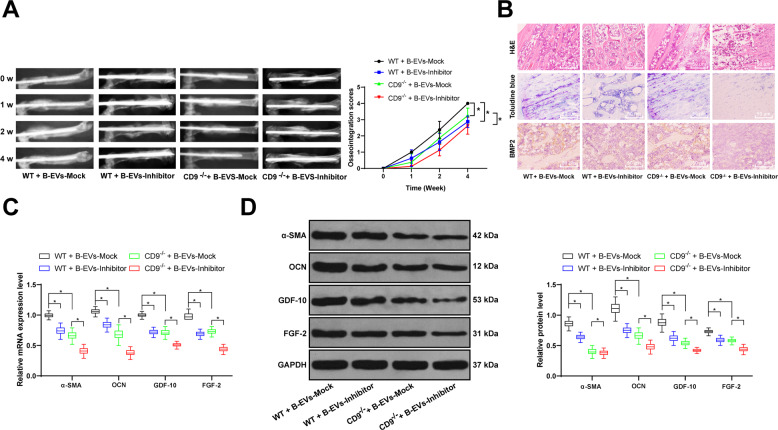
Silencing miR-335 partially counteracts the promoting effect of B-EV on fracture recovery. The EVs were extracted from BMSCs transfected with miR-335 inhibitor or its negative control Mock for 48 h, and treated on WT and CD9^−/−^ fracture mice on the 1st and 8th days after the fracture. **A** Representative radiologic images of WT and CD9^−/−^ fracture mice at 0, 1, 2, and 4 weeks after EV treatment. **B** HE staining, Toluidine blue staining, immunochemistry of BMP2 were performed to observe the femoral fracture healing of WT and CD9^−/−^ fracture mice treated with EVs-inhibitor. **C** RT-qPCR and **D** western blot analysis were performed to determine mRNA and protein levels of α-SMA, OCN, GDF-10, and FGF-2 in femurs of mice 2 weeks after fracture. Data are expressed as mean ± standard deviation. Two-way ANOVA and Tukey’s multiple comparisons test were used to determine statistical significance. *p < 0.05. *n* = 8 in each group.

From the above results, we found that 2#-Inhibitor had the best inhibitory effect on miR-335, so we extracted the B-EVs treated with 2#-Inhibitor or Mock to treat WT and CD9^−/−^ fracture mice. The results of X-ray showed that the fracture healing of EVs-inhibitor group was significantly lower than that of EVs-mock group (Fig. 3A). HE staining, toluidine blue staining and BMP2 immunohistochemistry showed that EVs-inhibitor reduced the formation of bone and cartilage tissues, and significantly reduced the levels of osteoblast differentiation (Fig. 3B). RT-qPCR and western blot results showed that the mRNA and protein levels of α-SMA, OCN, GDF-10, and FGF-2 were significantly decreased (Fig. 3C–D). The above results suggested that the intervention of miR-335 transported by B-EV partially counteracted the promoting effects of B-EV on fracture recovery in mice.

### B-EV treatment promotes osteoblast differentiation

**Fig. 4:**
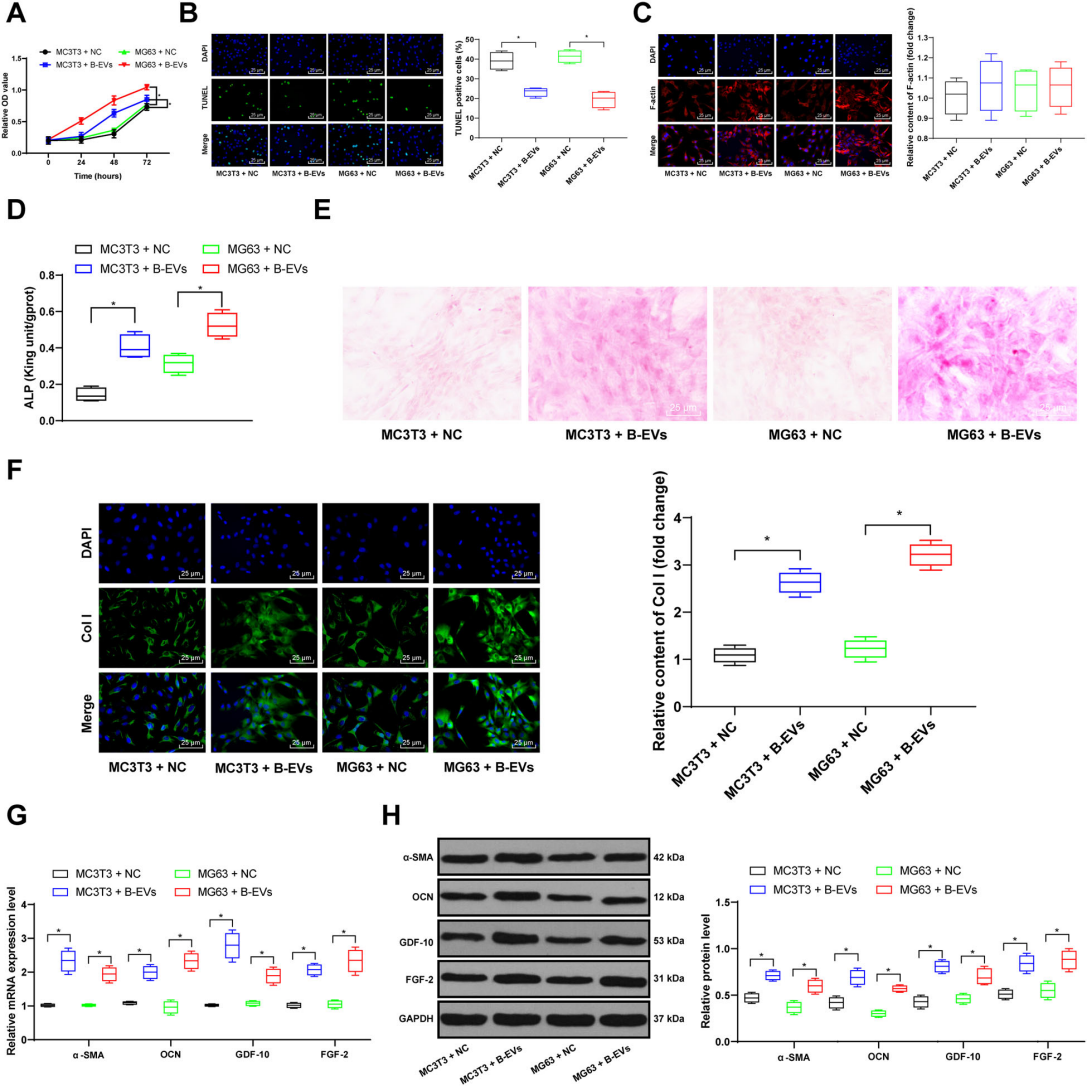
B-EVs promote osteoblasts-like cell differentiation. The EVs were extracted from BMSCs and treated on MC3T3 and MG63 cells for 24 h, respectively. **A** MC3T3 and MG63 cell viability was determined by MTT assay. **B** TUNEL staining was performed to determine apoptosis of MC3T3 and MG63 cells. **C** Immunofluorescence staining measured the expression of F-actin in cells. **D** ALP activity in MC3T3 and MG63 cells was determined by ALP assay. **E** Alizarin red staining was performed to validate MC3T3 and MG63 bone differentiation ability. **F** Immunofluorescence staining measured the expression of Col I in cells. **G**, **H** RT-qPCR and western blot analysis were performed to determine mRNA and protein levels of α-SMA, OCN, GDF-10, and FGF-2. Data are expressed as mean ± standard deviation. Two-way ANOVA and Tukey’s multiple comparisons test were used to determine statistical significance. *p < 0.05. Four independent experiments were performed.

**Fig. 5:**
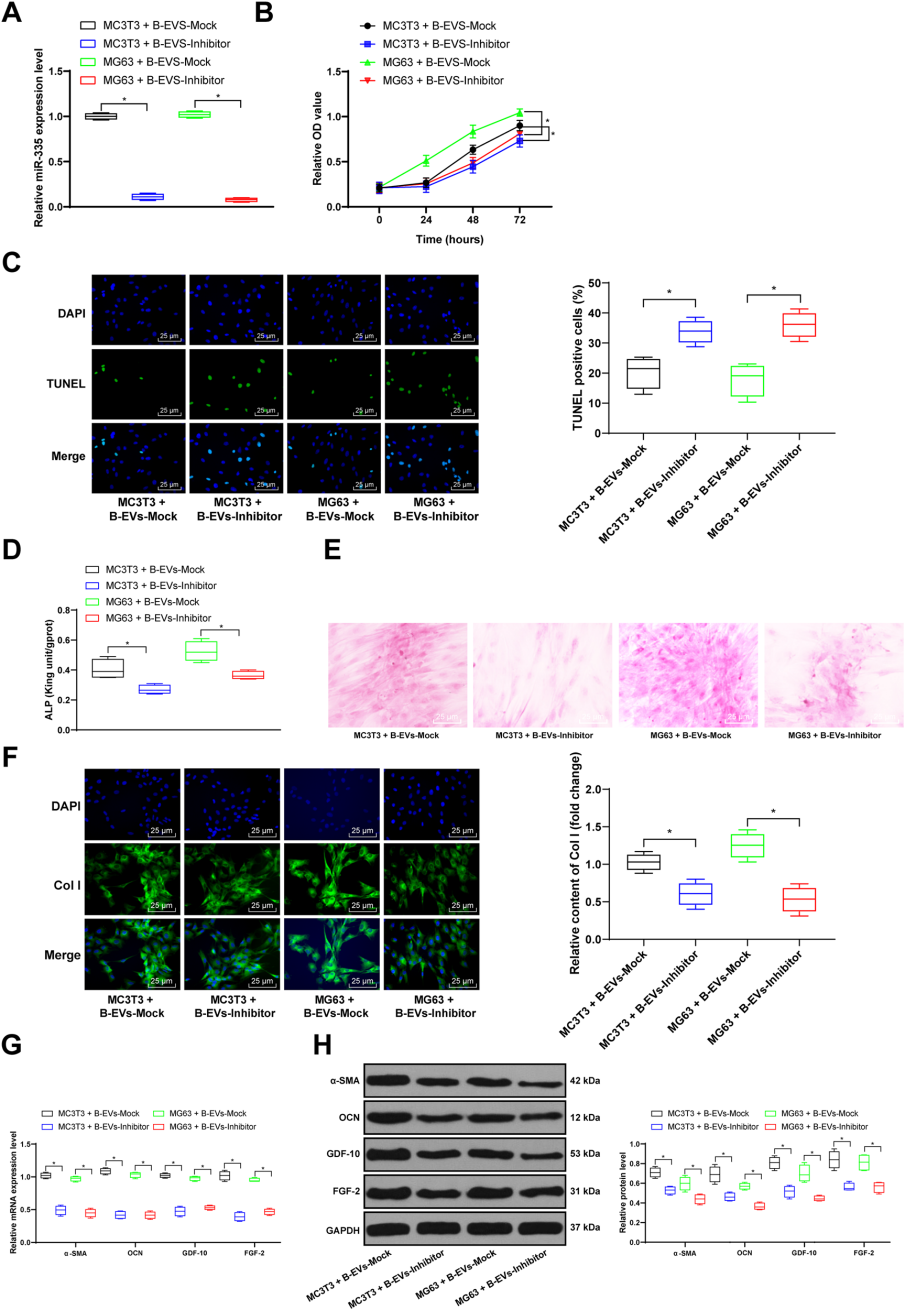
Inhibition of miR-335 attenuates B-EV effects. The EVs were extracted from BMSCs transfected with miR-335 Inhibitor or Mock for 48 h, and treated on MC3T3 and MG63 cells for 24 h. **A**–**B** MC3T3 and MG63 cell viability was determined by MTT assay. **C** TUNEL staining was performed to determine apoptosis of MC3T3 and MG63 cells. **D** ALP activity in MC3T3 and MG63 cells was determined by ALP assay. **E** Alizarin red staining was performed to validate MC3T3 and MG63 bone differentiation ability. **F** Immunofluorescence staining measured the expression of Col I in cells; **G**, **H** RT-qPCR and western blot assays were performed to determine mRNA and protein levels of α-SMA, OCN, GDF-10, and FGF-2. Data are expressed as mean ± standard deviation. Two-Way ANOVA and Tukey’s multiple comparisons test were used to determine statistical significance. *p < 0.05. Four independent experiments were performed.

The above results indicated that EVs carrying miR-335 has a positive effect on fracture recovery and can promote the differentiation of osteoblasts. To deeply discuss the mechanism of B-EVs in fracture recovery, we further verified the effect of B-EVs on osteoblasts in osteoblasts-like cells MC3T3 and MG63. First, MTT method detected the proliferation rate of MC3T3 and MG63 cells, and found that the treatment of EVs promoted cell proliferation (Fig. 4A). The results of TUNEL staining showed that EV treatment inhibited apoptosis (Fig. 5B). Subsequently, we observed cell adhesion by immunofluorescence staining of F-actin in MC3T3 and MG63 cells, and found that B-EV treatment had no significant effect on cell adhesion (Fig. 4C). ALP expression in MC3T3 and MG63 cells was detected by ALP kit, and it was found that B-EV treatment promoted ALP expression (Fig. 4D). In addition, alizarin red staining indicated that B-EV treatment promoted osteoblast differentiation and formed more bone nodules (Fig. 4E). The immunofluorescence staining of Col I (a protein associated with cellular osteogenic differentiation and promoting osteonodule) demonstrated that B-EVs promoted Col I expression, consistent with alizarin red staining results (Fig. 4F). RT-qPCR and western blot analysis verified that B-EV treatment increased levels of α-SMA, OCN, GDF-10, and FGF-2 (Fig. 4G–H).

### Silencing miR-335 reverses the promoting effect of B-EVs on osteoblast differentiation

In vivo experiments, we found that inhibition of miR-335 expression in EVs weakened its effect on fracture recovery. To explore its mechanism, we also observed in MC3T3 and MG63 cells. To investigate miR-335 functions in osteoblast differentiation, the EVs from BMSC transfected with 2#-Inhibitor and corresponding NC Mock were added to MC3T3 and MG63 cells. RT-qPCR showed miR-335 expression was decreased significantly after EVs-Inhibitor treatment, accompanied by a decrease of cell proliferation rate and an increase of cell apoptosis (Fig. 5A–C). Moreover, ALP, alizarin red and Col I immunofluorescence staining revealed that the intervention of miR-335 carried by B-EVs partially counteracted the promoting effects of B-EVs on osteoblast differentiation of MC3T3 and MG63 cells (Fig. 5D–F). The mRNA and protein levels of α-SMA, OCN, GDF-10, and FGF-2 were significantly decreased after EVs-inhibitor treatment (Fig. 5G–H).

### miR-335 could target VapB

**Fig. 6:**
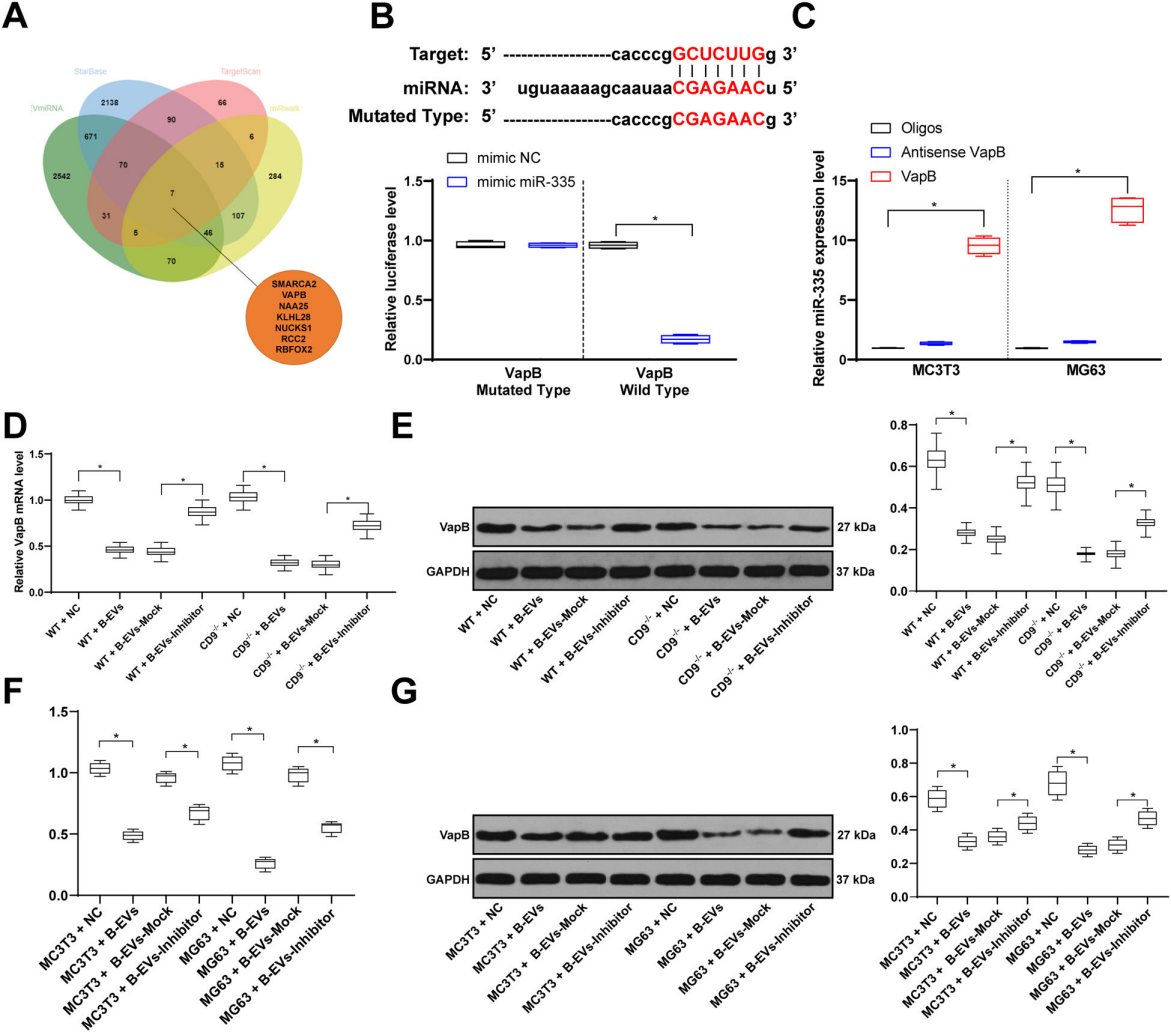
miR-335 promoted bone fracture recovery via targeting VapB. **A** EvimicroRNA and Starbase predicted miR-335 target gene, and seven mRNAs were selected. **B** dual-luciferase reporter gene assay in HEK293T cells and **C** RNA pull-down assay were performed to validate the combination between miR-335 and VapB in MC3T3 and MG63 cells. VapB mRNA and protein levels in fracture mouse model (**D**, **E**) or MC3T3 and MG63 cells (**F**, **G**) were determined by RT-qPCR and western blot analysis. Data are expressed as mean ± standard deviation. Two-way ANOVA and Tukey’s multiple comparisons test were used to determine statistical significance. *p < 0.05. Four independent experiments were performed.

In vivo and in vitro experiments have confirmed that B-EVs could promote fracture recovery by carrying miR-335. In order to further clarify the downstream mechanism of miR-335, we predicted and screened seven target genes (SMARCA2, VAPB, NAA25, KLHL28, NUCKS1, RCC2, and RBFOX2) of miR-335 on the website of miRwalk, TargetScan, EvimcrRNA, and Starbase (Fig. 6A). Through a literature review, we found that VapB could promote the growth of osteoclasts^33^, so we focused on VapB. The dual-luciferase reporter gene assay in HEK293T cells^34^ showed that miR-335 could target VapB, and then the Biotin-labeled RNA pull-down assay verified that miR-335 could target VapB in MC3T3 and MG63 cells (Fig. 6B–C). RT-qPCR and western blot analysis detected VapB levels in mice and cells. The results showed that EV treatment inhibited VapB levels, whereas miR-335 inhibition could alleviate the inhibition of VapB (Fig. 6D–G).

### VapB overexpression reverses the promoting effect of B-EVs on osteoblast differentiation

**Fig. 7:**
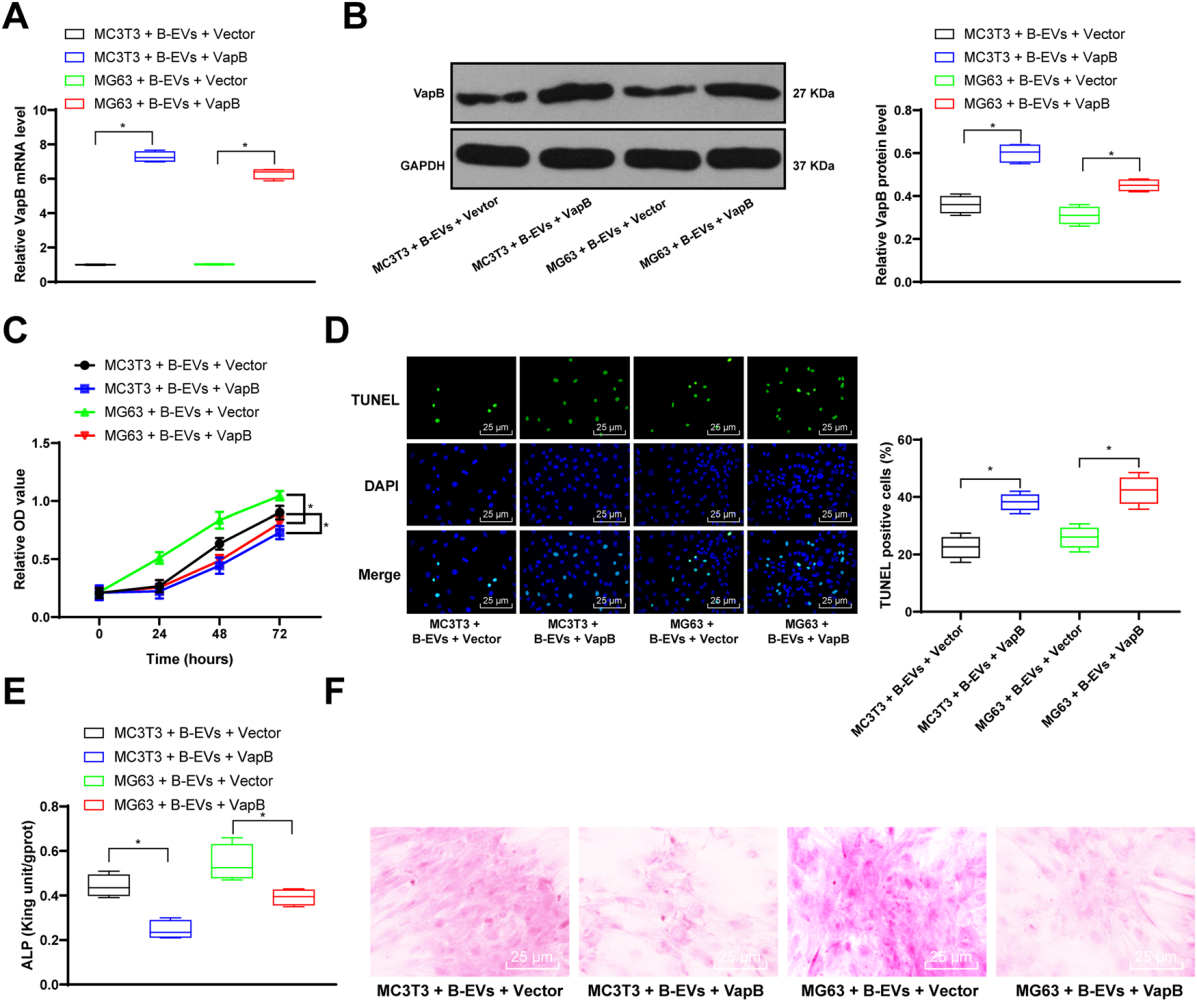
VapB overexpression attenuates B-EV effects. The EVs were extracted from BMSCs and treated with VapB overexpression plasmids on MC3T3 and MG63 cells. And the control was cells were treated with B-EVs and Empty Vector. **A**, **B** RT-qPCR and western blot analysis were performed to determine VapB mRNA and protein levels. **C** MC3T3 and MG63 cell viability was determined by MTT assay. **D** TUNEL staining was performed to determine apoptosis of MC3T3 and MG63 cells. **E** ALP activity in MC3T3 and MG63 cells was determined by ALP assay kit. **F** Alizarin red staining was performed to validate MC3T3 and MG63 bone differentiation ability. Data are expressed as mean ± standard deviation. Two-way ANOVA and Tukey’s multiple comparisons test were used to determine statistical significance. *p < 0.05. Four independent experiments were performed.

To investigate VapB functions in osteoblast differentiation, the B-EVs-treated cells were transfected with VapB overexpressing vectors and corresponding empty vector. RT-qPCR showed that VapB expression in cells was increased significantly after transfection, accompanied by the decrease of cell proliferation rate and the increase of cell apoptosis; while VapB overexpression induced cell apoptosis (Fig. 7A–D). Moreover, VapB overexpression partially counteracted the promoting effects of B-EVs on osteoblast differentiation of MC3T3 and MG63 cells (Fig. 7E–F).

### B-EVs-carrying miR-335 targets VapB to activate the Wnt/β-catenin pathway

**Fig. 8:**
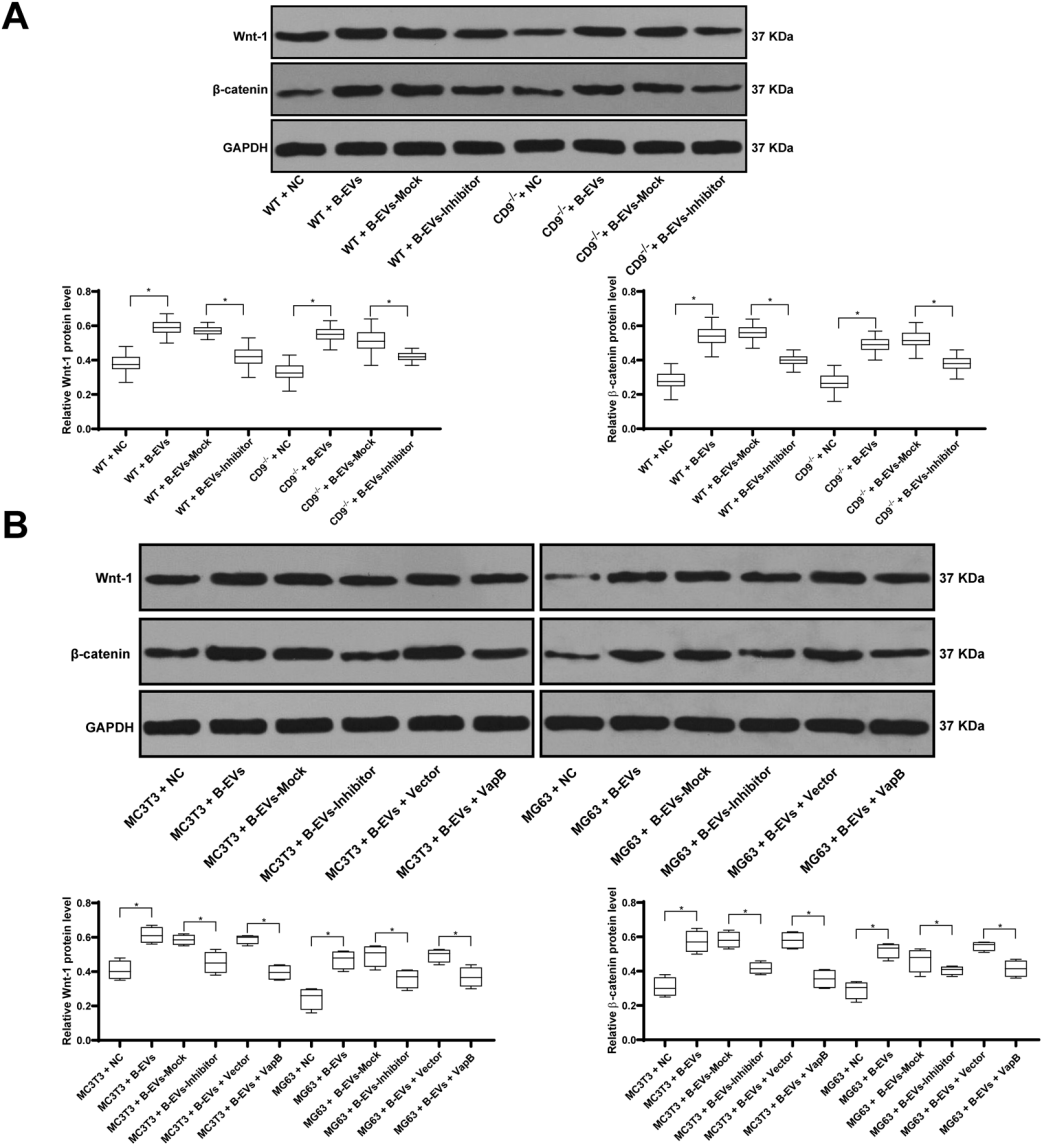
B-EVs-carried miR-335 targets VapB and promotes bone fracture recovery by activating the Wnt/β-catenin pathway. WT or CD9^−/−^ mice 2 weeks after fracture were treated with B-EVs or B-EVs-Inhibitor on the 1st and 8th days. B-EVs treated mice or MC3T3 and MG63 cells were transfected with the VapB overexpression plasmid for 24 h. **A**, **B** Western blot analysis was performed to determine Wnt/β-catenin-related protein levels in femurs of fracture mice and MC3T3 and MG63 cells. Data are expressed as mean ± standard deviation. One-way ANOVA and Tukey’s multiple comparisons test were used to determine statistical significance. *p < 0.05. *n* = 8. Four independent cell experiments were performed.

Wang Y et al.^35^ has reported that Wnt/β-catenin pathway can promote osteogenic differentiation in mice. In a report published by Kramer et al., downregulation of β-catenin can lead to increased bone resorption^36^. In the above results, we found that EVs can promote osteogenic differentiation. To thoroughly investigate the mechanism of EVs, we detected the levels of Wnt/β-catenin pathway in fracture mice and osteoblast-like cells. It was found that levels of Wnt/β-catenin pathway were increased significantly after B-EV treatment, but decreased after EVs-Inhibitor treatment (Fig. 8A). In the previous results, we found that miR-335 has binding sites with VapB, and overexpression of VapB inhibited the differentiation of osteoblasts. Therefore, we speculated that the expression of VapB may be related to Wnt/β-catenin pathway. As showed in Fig. 8B, overexpression of VapB reversed the promotion of Wnt and β-catenin protein expression by EVs. These results suggested that miR-335 targeting VapB in EVs may be related to Wnt and β-catenin pathways.

## Discussion

Fracture recovery is a complicated biological process that requires interactions among different cell types, including cells in the periosteum, stem/progenitor cells, and chondrocytes into osteoblasts^37^. MSCs from the systemic circulation are thought to migrate to the damaged site and lead to bone formation at the fracture site^38^. In this study, we evaluated the effects of B-EVs and the molecular mechanisms in fracture recovery. As expected, our results supported the conclusion that B-EVs carry miR-335 to osteoblasts-like cells to upregulate miR-335 expression and inhibit VapB expression. The decreased VapB expression promoted the activation of Wnt/β-catenin pathway, increased the content of α-SMA, OCN, GDF-10, and FGF-2, and facilitated osteogenic differentiation and fracture healing (Supplementary Fig. 2).

To begin with, HE staining, toluidine blue staining, and immunohistochemistry of BMP2 showed that B-EV treatment promoted the formation of bone tissues and cartilage tissues, osteoblast differentiation, and fracture recovery. Similarly, in the collagenase-induced osteoarthritis mouse model, microparticles and exosomes isolated from BMSCs can effectively protect cartilage and bone from degradation^39^. In addition, B-EVs increased ALP expression and levels of α-SMA, OCN, GDF-10, and FGF-2. ALP is an early marker of osteogenesis, OCN is a late osteogenic marker, and calcified nodules are signs of the final stage of osteogenesis^40,41^. Fracture recovery and endochondral bone formation are regulated by BMPs, FGF-2, and Wnt proteins^1^. MSCs have the potential to reduce healing time and treat nonunion in fracture patients and to promote callus formation by expressing BMP2^42^. Fracture or injury initiates a series of cellular and molecular pathways that modulate MSCs activity from hematoma formation and inflammation, leading to fracture healing and bone integrity reconstruction^43^. Similarly, human umbilical cord-MSCs-exosomes stimulate cell proliferation, and inhibit cell apoptosis and inflammatory response, thus inducing tissue regeneration^44^. B-EVs attenuated radiation-induced bone loss, diminished oxidative stress, stimulated DNA damage repair, and kept the balance between adipogenic and osteogenic differentiation^45^. In summary, B-EV treatment promoted the fracture recovery in mouse model and enhanced osteoblast differentiation in cell models.

To further determine the specific mechanism of EV treatment in fracture recovery, differentially expressed miRs in B-EVs-treated C57BL/6 WT mice were screened via microarray analysis and verified by RT-qPCR. It was found that miR-335 was abundant in MSCs and B-EVs. miR-335-5p is highly abundant in undifferentiated MSCs and osteoblasts in case of bone development, and its neutralization in bone marrow mononuclear cells transplanted into a large femur defect of the rat improved bone healing^46,47^. miR-335 is recently verified as the most upregulated miR in BMSCs relative to skin fibroblasts^48^. Importantly, miR-335-5p-modified BMSCs in osteoblast lineage stimulated osteogenic differentiation and bone formation in mice, and increased levels of osteogenic transcription factors^49^. In addition, we found that intervention of miR-335 carried by B-EVs partially counteracted the promoting effects of B-EVs on fracture recovery in mice, and osteoblast differentiation of MC3T3 and MG63 cells. Osteoblasts are of prime importance in the initial bone formation and fracture repair, and increasing MSC or differentiation factors at the fracture sites are capable to induce MSC differentiation into osteoblasts^50^. miR-335-5p was suggested as an excellent indicator of fracture patients with low-trauma^51^. Furthermore, we verified that miR-335 could target VapB, and VapB overexpression reversed the promoting effect of B-EVs on osteoblast differentiation. VapB expression presented an increase during osteoclast formation, and VapB knockdown led to repressed bone resorption^33^.

Moreover, we found the activity of Wnt/β-catenin pathway was increased significantly after B-EV treatment. A literature review notes that some Wnts shift cells towards the osteoblastic lineage by inducing osteoblast-related genes and suppressing adipogenic transcription factors, and activation of Wnt pathway stimulates osteoblast formation^1^. Similarly, miR-335-5p has been documented to activate the Wnt pathway to promote osteogenic differentiation by downregulating Wnt antagonist Dickkopf-related protein 1^52^. Wnts inhibited MSCs adipogenesis from undergoing adipogenesis and stimulated osteogenesis via a β-catenin-dependent manner^53^. B-EVs alleviate radiation-induced bone loss by restoring the function of recipient BMSCs and activating the Wnt/β-catenin pathway^45^. Meanwhile, overexpression of VapB inactivated the Wnt/β-catenin pathway and inhibited osteogenic differentiation.

There are several limitations of this study. First, owing to the limitations of experimental conditions, among the eight differentially expressed miRs, we only selected miR-335 with the highest differential expression. The other seven miRs also play critical roles in osteogenic differentiation of MSCs^54–60^, which suggests that multiple miRs may play a role in the synergistic miR-miR interaction network in the process of fracture repair^61,62^. Next, we will continue to pay attention to the role of these differentially expressed miRs. Second, the specific mechanism of miR-335 and VapB in apoptosis is not specifically discussed in this study, but this is an interesting research direction. In the following study, we will conduct a more in-depth study on the mechanism. Third, owing to the experimental conditions and funds, we did not study other sources of MSCs. But based on other studies^63–65^, we speculated that MSCs from other sources such as adipose or umbilical cord could promote bone healing, but this still needs to be confirmed in subsequent experiments.

In summary, our results supported that B-EVs promotes osteoblast differentiation and bone fracture recovery. Of particular note, exosomal miR-335 could promote bone fracture recovery by activating the Wnt/β-catenin pathway by targeting VapB. The study yields novel insights into the exosomal circulating miR for therapeutic approaches in bone fracture treatment. B-EVs-based therapy may be an application focus for clinical treatment. We hope this study can provide new perspective for bone fracture molecular mechanism and offer novel insights for bone fracture treatment from the aspect of B-EVs-based therapy.

## Supplementary information

Supplementary Figure 1

Supplementary Figure 2
