# Supplementary figures and images for: Role of microRNA-335 carried by bone marrow mesenchymal stem cells-derived extracellular vesicles in bone fracture recovery
Source: Cell Death Dis. 2021 Feb 4;12(2):156. doi: 10.1038/s41419-021-03430-3 (PMC7862274; doi:10.1038/s41419-021-03430-3)

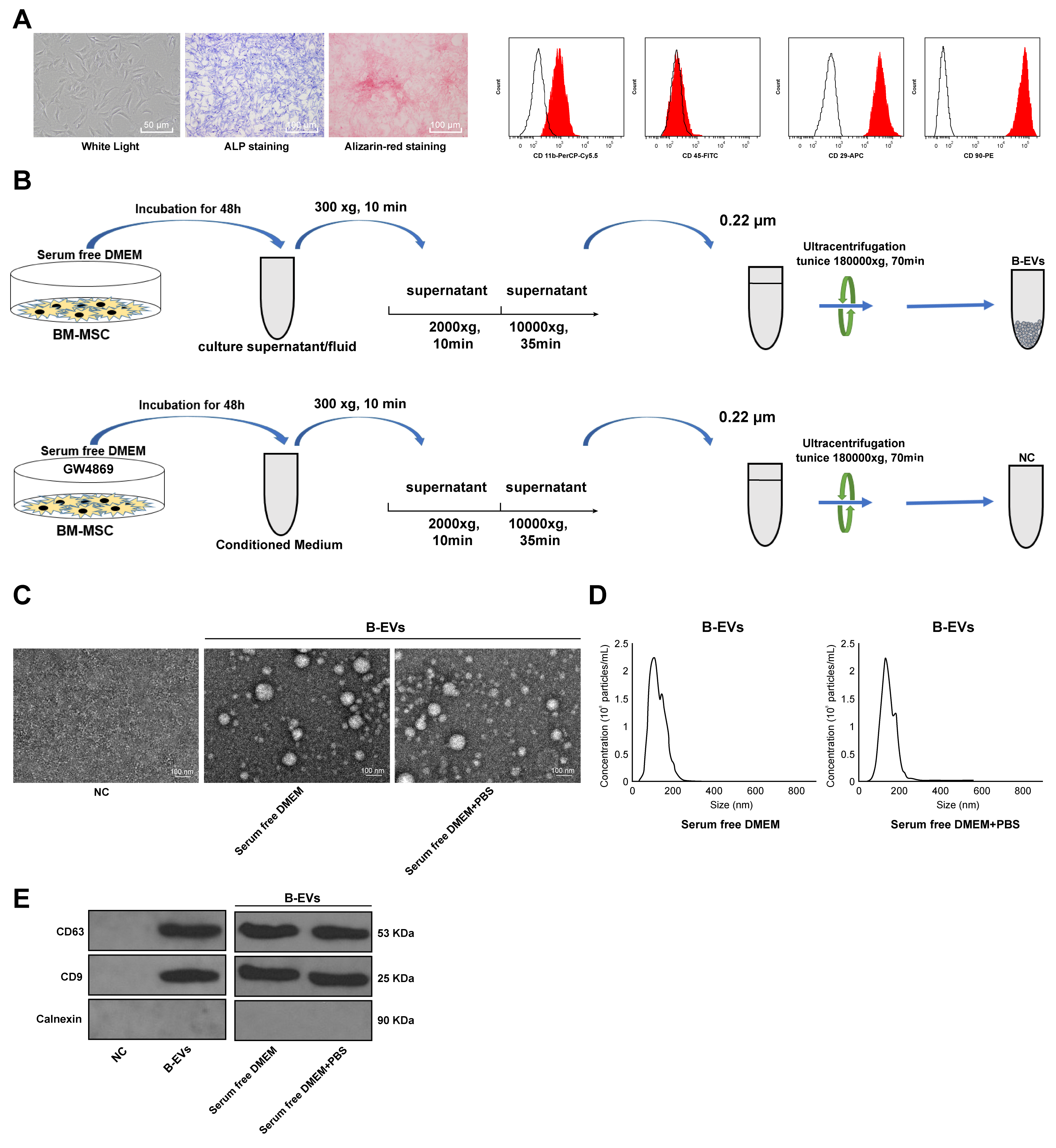

Supplement: Supplementary file 1 — Supplementary Figure 1 [file 41419_2021_3430_MOESM1_ESM.tif]

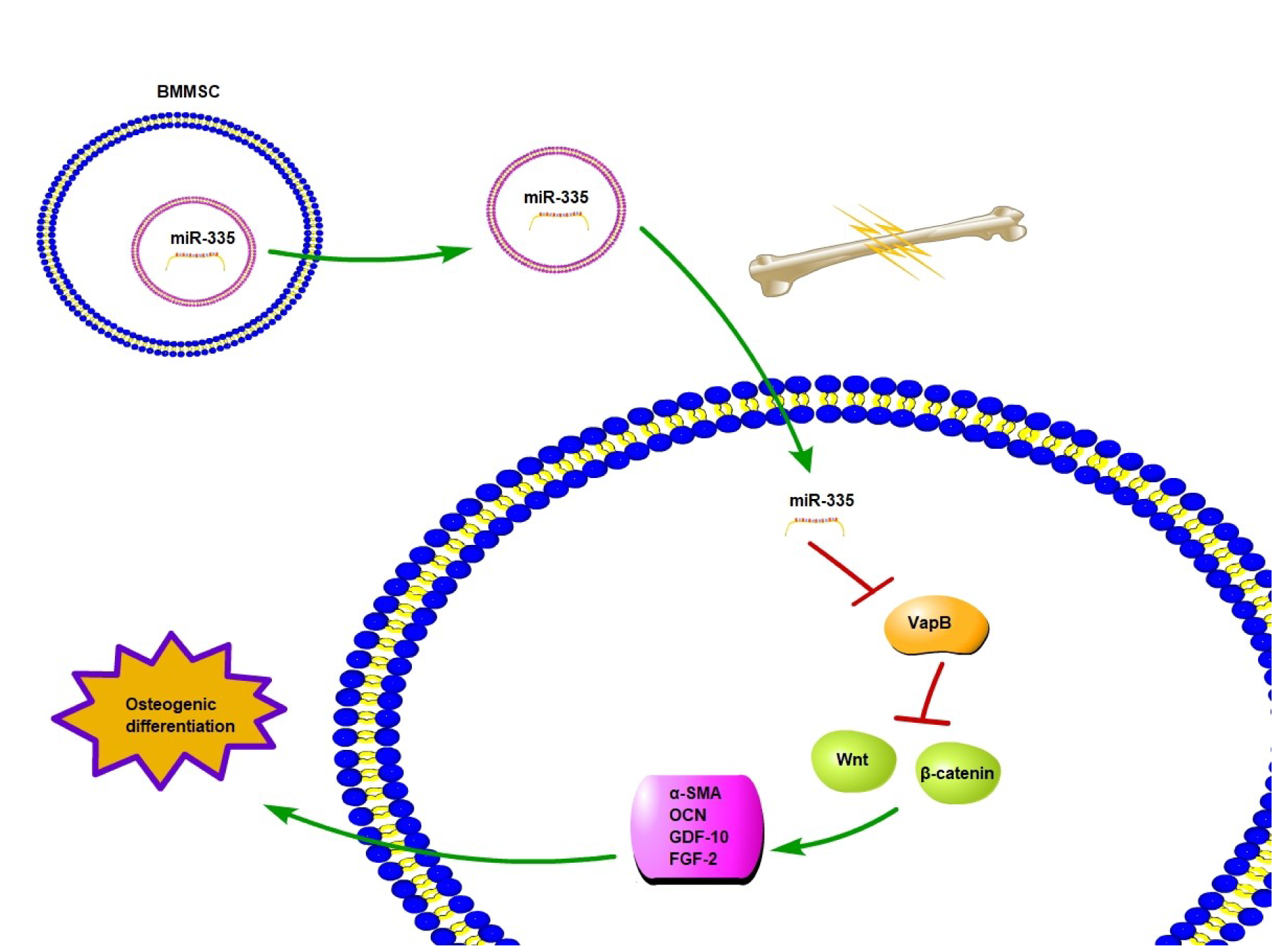

Supplement: Supplementary file 2 — Supplementary Figure 2 [file 41419_2021_3430_MOESM2_ESM.tif]
